# The Diagnosis of the Internal Secretory Diseases

**Published:** 1916-11

**Authors:** Henry R. Harrower

**Affiliations:** M. D., F. R. S. M. (Lond.), Los Angeles, California


THE
### TEXAS MEDICAL JOURNAL
Dr. F. E. Daniel, Founder. Established July, 1885
MRS. F. E. DANIEL, - Publisher and Managing Editor
Published Monthly.—Subscription, $1.00 a Year.
VOL. XXXII AUSTIN, NOVEMBER, 1916 No. 5
The publisher is not responsible for views of contributors

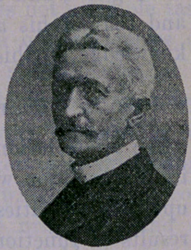

*"All of good the past hath had,
Remains to make our own time glad."*—WHITTIER.

### Original Articles.

### The Diagnosis of the Internal Secretory Diseases.

BY HENRY R. HARROWER, M. D., F. R. S. M. (LOND.), LOS ANGELES, CALIFORNIA.

### VI.

### THE DISORDERS OF THE PITUITARY BODY.

The study of the various phases of endocrinology seems to have advanced in waves; and our knowledge of the clinical and physiological relations of the hypophysis of pituitary body is a good example of this. Thirty years ago quite an interest was aroused in this remarkable gland by the publication of Marie's classical study of the pathology of acromegaly and his correlation with it of disease of the pituitary gland. Nearly ten years later—in 1894—a greater wave of enthusiasm and interest was launched by Sir Edward A. Schaefer, who made the discovery that the pituitary was a gland of internal secretion. Numerous investigations were initiated by this report, many of which have added materially to our knowledge of this subject.

The third and greatest wave of all must be connected with the name of Harvey Cushing, and this has brought us to the present high tide of knowledge of the subject, for, thanks to the results of the years which Cushing has spent in investigating pituitary disorders, the profession is better able to realize the comparative frequency of affections of this gland.

Cushing's monograph, "The Pituitary Body and Its Disorders"

(1912) has been called the most complete and useful monograph in English; and the numerous publications of reports of his work and that of his associates include the major part of our present knowledge on this subject.

PHYSIOLOGICAL CONSIDERATIONS.

An appreciation of the essentials of the physiology of this gland, its interrelation with the other endocrine organs, and its influence upon the activities of the body, will enable us to detect the several results of functional pituitary dyscrasia during their early stages, before such obvious and serious changes as those present in acromegaly have established themselves.

It must be recalled that structurally the pituitary gland is divided into three parts: The largest anterior lobe being a typical glandular structure; the much smaller posterior lobe having the histological appearance of nervous tissue, while the very small connecting portion, usually called by its Latin name, "pars intermedia," is made up of a mixture of both kinds of these cells. Each of these portions produces one or more chemical substances or hormones, the functions of which are not all fully understood. Without going into detail, it may be stated that the anterior lobe produces a hormone which regulates the growth of the body. This was isolated recently by T. Brailsford Robertson of the University of California, and has been called by him "tethelin." In both physiology and organotherapy this substance promotes growth, especially that of bone and connective tissue; and it is anticipated that many useful advances in organotherapy will follow the clinical experimental study of preparations derived from the anterior lobe of the pituitary.

From the posterior lobe there is secreted, presumably directly into the cerebro-spinal canal, a series of hormones, which play an important part in the control of metabolism, especially that of the carbohydrates. They also influence in some subtle way the sympathetic nervous system quite similarly to the chromaffin hormone from the adrenals. Much clinical use has been made of the extract of the posterior lobe, and it undoubtedly exerts a very wonderful pharmacological influence upon unstriped muscle and particularly upon the uterus in labor. A diuretic hormone of considerable activity is also produced in this gland, some saying that it arises in the pars intermedia and others in the posterior lobe.

The pituitary body as a whole is very intimately connected with sex development, as we shall shortly see; and is able to assist the thyroid and gonads vicariously when this becomes necessary. These complex relationships complicate the study of the subject, and it might just as well be stated right here that it is not a simple task accurately to differentiate between the results of deficiencies of these endocrine glands, for their relations are so intimate that it is quite impossible for one to be affected without some chemical reflex influence being brought about in the work of most or all of the others; and as these glands seem to exert a compensatory influence upon the work of those glands with which they are correlated, it is often difficult to determine the original gland at fault in a given case, and unless this is done, suitable treatment, organotherapeutic or otherwise, may be impossible.

We have just noted that there is a great functional difference between the parts of the pituitary. Like a number of other endocrine organs, it is a dual one, with differing structure and physiological powers; and it is possible that clinical manifestations due to affections of one lobe may differ very materially from those due to disturbances of the other. An attempt to facilitate a differentiation between the disorders of the two lobes will follow the consideration of disease of the whole gland.

### DYSPITUITARISM.

When disorders of the pituitary gland are the result of tumors, cysts or intracellular dyscrasia, there may be varying secretory changes. On the one hand pressure due to the growth may prevent the normal secretory activity, while, on the other, the enlargement may be a pure hyperplasia with markedly increased function until the limitations of the sella turcica—the bony cup above the splenoid bone in which the pituitary rests—cause a secondary hypofunction. Such cases are termed dyspituitaric, since varying results are produced. In fact, many individuals suffering from pituitary excess have at the same time well defined evidences of pituitary insufficiency, secondary to the original trouble.

Dyspituitarism, then, is pituitary secretory dyscrasia and may include the pure hypo and hyper-function and all grades between them and combinations of them. By careful study it is often possible to decide which disturbance is predominant and also which is the original disorder. A diagnosis of "dyspituitarism" is good; but to qualify this and go further into the genesis of the disorder, is much better.

PITUITARY INSUFFICIENCY.

With the fundamentals previously outlined in mind, we can expect marked changes in the metabolism as a result of insufficient activity of the pituitary gland. The most common result of insufficient function—hypopituitarism—is an undue increase in the deposit of fat which may later become a serious obesity, a condition which is probably due to the marked increase in the tolerance for carbohydrates usually found in hypopituitarism,* and the abnormal desire for food and especially for sweets with which this is quite often associated. It is not uncommon to find patients in this class eating ravenously with appetites far beyond the usual.

The cellular activities are generally reduced and the temperature is subnormal, movements slow and somnolence is a prominent symptom. Cushing and his associates have remarked that hibernation in certain animals seems to be a physiological hypopituitarism. Lassitude, torpidity and drowsiness is often the first appreciated symptom. (Some months ago I saw a case with Dr. Roblee at Riverside, who would fall asleep during meals or in the middle of a sentence; and who, by the way, improved very much under pituitary medication.) Sleep is not always refreshing and tiredness is a usual complaint.

This reduced oxidation is probably due in part to an associated thyroid insufficiency. The urinary solids are reduced, but the amount of urine is often increased; and it is now believed that the majority of those suffering from extreme polyuria, or diabetes insipidus, really have a form of pituitary disease. According to Motzfeldt and others, the lesion is in the posterior lobe, and the functional changes are on the side of hyposecretion.

There are retrogressive changes in the sex organs and functions. The syndrome described by Froelich and Bartels—the so-called "dystrophia and adiposo-genitalis"—is due to hypopituitarism, the adiposity being marked and the sex changes characteristic.

The age at which these conditions assert themselves naturally causes variations in the manifestations. When pituitary insufficiency is present in childhood or early youth, the developmental

*A urinary test for dyspituitarism is thus made possible. The high tolerance for sugar is usual in hypopituitarism. This may be easily demonstrated by giving measured, increasing amounts of sugar or, preferably, levulose, and noting how much may be taken without glycosuria. Often as much as 250 grams can be eaten (Cushing reports a case in which 450 grams were taken) without a trace of glucose in the urine passed during the next few hours thereafter. On the other hand in the opposite secretory condition—hyperpituitarism—there is a very low sugar tolerance, and not infrequently there may be glycosuria.

changes are more marked. The stature is small and skeletal growth is stunted. Genu valgum is quite common. The fingers are frequently tapered, and acromicria, i. e., unusually small hands and feet, has been noted by Timme, though this is rare compared with the corresponding opposite (acromegaly) in the opposite condition. The epiphyses may remain ununited and, parenthetically, it is well in cases of reduced stature to have roentgen pictures made of a hand, so that if defective epiphysial growth is present, there is hope for comparatively successful results from suitable organotherapy. On the other hand, in dwarfs showing fully united epiphyses there is little hope that the most effective therapeutic measures will increase the stature.

Temperamentally hypopituitaric children are dull, apathetic, backward in their studies and easily discouraged. They often have difficulties with their playmates and lack both self-reliance and self-control.

The abnormalities of sex development are among the most typical results of pituitary insufficiency. The external genitals are small, the pubertial growth of hair is sparse or absent. There may be either cryptorchism or infantile uterus with impotence or amenorrhea. The menses appear late or not at all, and if amenorrhea is not complete, the flow is scanty and irregular. The breasts often become extremely large, due both to the adiposity usually present and to the reduced gonad activity. A peculiar and quite constant finding is a tendency to development which simulates that of the opposite sex, especially in the male, in which the pubic hair line is straight and the contour of the hips and chest quite female in type.

The head is often small and the face unintelligent, and the distance between the eyes narrowed. The teeth are usually malformed and broad. The skin is dry and soft, and, compared with the dry, rough skin of hypothyroidism, is quite smooth to the touch. Perspiration is much reduced, even in hot weather and during exertion.

When hypopituitarism is acquired after maturity it is often the result of syphilis, and the developmental changes just enumerated are not present. Here, however, there is anaphrodisia and sexual atrophy, obesity which may be extreme, with difficulty in locomotion and work, with a natural tendency to laziness and lethargy, which further increases the asthenia and deposition of fat. Occasionally the fatty deposits are painful on pressure and are very similar to Dercum's disease or adiposis dolorosa, a condition which is probably of both pituitary and thyroid origin. This adiposity causes difficulties with the heart and breathing and edema may supervene due to fatty pericardial involvement.

Asthenia is the rule, irrespective of the extent of the obesity, and the unstriped muscles seem to be affected equally with the voluntary muscles, hence constipation is common and the bladder walls may be unduly weak with incontinence. The heart action is weak and the pulse slow and of reduced volume. The blood pressure is low, ranging from 100 mm. Hg. to as low as 50 mm. or less. The circulation is poor, the extremities are cold and sometimes edematous late in the day, and occasionally the skin exhibits the mottled appearance referred to in the previous chapter.

Several authorities have noticed epilepsy as an accompaniment of hypopituitarism. Just what is the relationship we have yet to learn, but several writers, including Cushing, have remarked that pituitary feeding caused a decided benefit to the epileptic manifestations as well as those which are more generally recognized as of pituitary origin.

### HYPERPITUITARISM.

The start toward our present knowledge of the conditions associated with pituitary excess (hypertrophy and secretory activity) was made in the report of several cases in 1886 by Pierre Marie. He called the syndrome "acromegalia" because of the usually large hands and feet, which were a prominent part of the clinical syndrome. A comparison of the manifestations of increased pituitary secretion would be expected to show diametrically opposite findings to many of the hypopituitaric conditions above. For example, children with hyperpituitarism are large for their age, tall and bony framed. Their eyes are wide apart, the face is broad, the cheeks prominent and the jaw square and large. The condition of the facial bones is generally called prognathism. The teeth are many times large, broad and irregularly spaced.

Such individuals have large hands and feet, with long fingers and toes and an unusually early epiphyseal union. The hair is usually profuse, exhibits a tendency to grow low on the forehead, well up on the abdomen and, occasionally, hypertrichosis is present. The axillary and pubic hair comes unusually early and is always excessive. The skin is thick, harsh and sometimes puffy. The sweat glands are usually active.

The sexual development is excessive and in early cases precocity is to be expected and sexual irritability marked. The sympathetic system is well developed and highly sensitive. Hyperpituitaric individuals are often bright and keen and very excitable, though they lack the power of concentration and are indecisive. Temperamentally they are often irritable, distrustful, petulant and "difficult." They do not sleep well and insomnia is progressive as the glandular hypertrophy causes the local symptoms which will be referred to shortly.

The metabolism is plus and much accumulation of fat is rare. There may be a slight increase in the temperature, and the urinary solids are often increased. The tolerance to carbohydrates is reduced and the "carbohydrate tolerance test" is positive with 25 or 50 grams of sugar, and not infrequently glycosuria is one of the symptoms of hyperpituitarism.

The pulse rate is occasionally increased, though not very rapid; but the blood pressure may be high, ranging from 150 to 180 or more.

A word or two about the relation of gigantism to acromegaly. Of course both these conditions are the result of hyperpituitarism; but in the former instance the dystrophy has commenced before ossification of the bones has taken place with a resultant increase in length principally. In acromegaly, i. e., hyperpituitarism after full development, the bone changes lean to thickness, hence the prognathism, protruding forehead and "heavy" facies, and the kyphotic spine not uncommonly seen.

### NEIGHBORHOOD SYMPTOMS.

When the secretory disturbances of the pituitary are coupled with hypertrophic changes, a series of localized symptoms are caused which are of a wholly distinct character from those due to chemical changes—the pressure or neighborhood symptoms. These are ultimate results and are practically always accompanied by changes in the size and conformation of the sella turcica, which can be seen and even measured by roentgenography.

Unfortunately, these pressure symptoms are often the first indication that we have dyspituitarism to contend with, and they are practically only seen in advanced cases. Under such circumstances we can expect to find supplementary evidence of the cause of the trouble by looking for the systemic chemical changes of pituitary origin. These have already been enumerated. These localized symptoms are often so serious as to call for cerebral decompression, and curative treatment is practically hopeless; while the general metabolic disturbances previously mentioned often may be favorably affected by persistent organotherapy.

To quote a statement from Cushing: "It is particularly important that we should learn to recognize these clinical expressions of hypophysial disorder in the absence of brain tumor symptoms or radioscopic enlargement of the pituitary fossa, in the same way that it is important for us to recognize thyroid disorders unaccompanied by gross evidence of change in the configuration of the gland."

Neighborhood symptoms may be roughly divided into two classes: Immediate (local) and intracranial (general) pressure effects. In the former we look for the results of pressure on the structures in contact with the mass, while in the latter those found in any brain tumor—due to the increased intracranial pressure.

Among the former symptoms are well marked eye symptoms, such as bitemporal hemianopsia (blindness of the outer temporal fields of vision) due to pressure on the optic chiasma. This usually first affects color only and later form. In more advanced cases, when the tumor extends beyond the sellar edges, squint results, due either to pressure on the sixth cranial nerve (internal strabismus) or the third cranial nerve (external strabismus). As a result of still more extensive involvement, there may be pressure on the crura cerebri and disturbances of gait with a positive Babinski sign. Certain epileptoid attacks, the so-called "uncinate fits" are occasionally seen and one probably due to pressure upon the uncinate gyrus. The relationship of epilepsy and pituitary disease is interesting and bids fair to offer a part of the solution of this problem.

Before the last of these pressure symptoms have been caused, general intracranial symptoms will have supervened. These consist chiefly of a severe intractable headache, paroxysmal in character and often affecting both temples, with vertigo, vomiting (often of the projectile type) and failing vision with later choked disc (papilloedema) and progressive destruction of the visual fields and ultimate optic atrophy.

### DIFFERENTIATING THE LOBES INVOLVED.

It is rare that we find dyspituitarism of a single lobe, though it is possible. It is not unusual, however, to find predominating symptoms indicating that the principal trouble is in one of the lobes. If we bear in mind the varying physiological activities of the different portions of the hypophysis we will expect to find anterior lobe disorders more frequently accompanied by changes in growth and skeletal development. We have seen that with hypersecretion early the result is giantism, whereas later in life acromegaly is the result. On the other hand, hyposecretion retards growth and if it comes early the result is infantilism, while later it brings about retrogressive changes in the sex organs and manifestations.

Dystrophies of posterior lobe origin are quite different, since they account for the metabolic changes which cause the adiposity and increased carbohydrate tolerance found in hypopituitarism, while the excessive secretory activity of the posterior lobe produces a relative carbohydrate intolerance with glycosuria and increased metabolism and loss of weight.

Commonly both lobes are affected simultaneously, though the effects of one lobe may be more prominent and may change at different stages of the disease. Froehlich's syndrome, for instance, is evidently due to a secretory deficiency of the whole gland.

### THE CAUSE OF PITUITARY AFFECTIONS.

Etiology is often of great service in making a therapeutically useful diagnosis. In the estimation of the writer syphilis is the chief cause of dyspituitarism, and while heredity is an evident factor, syphilis in parents and grandparents may have left an intangible susceptibility. The Wassermann test is very useful here.

New growths of the hypophysis, other than gummata, are common etiological factors, the causes of which are still altogether unknown. Early organic changes in the bony pituitary fossa may restrict the proper development of the growing gland. Brain tumors, either adjacent to the pituitary or remote from it, may cause dyspituitarism by increasing the intracranial pressure and the pituitary symptoms may entirely disappear following decompression.

It has also been suggested that as the posterior lobe is supposed to secrete into the cerebro-spinal canal, changes in the intraspinal pressure may cause pituitary disorder. The present epidemic of poliomyelitis in New York City should offer some proof of this position, for if this is true, there should be a moderately large percentage of pituitary disorders follow in the hundreds marked by this disease; for we know that poliomyelitis is often accompanied by increased intraspinal pressure and that the relief of this tension causes a favorable effect on some of the symptoms.

Next month, "Disturbances of the Thymus."

